# Upon a Singular Variety of the Human Species

**Published:** 1810-11

**Authors:** 


*For the Medical Journal.*

### *Upon a Singular Variety of the Human Species.*

THE annexed Plate, for which we are indebted to Mr. Tresham, is a tolerably correct representation of a female lately brought to London from the Cape of Good Hope, and now exhibiting under the appellation of "*The Hottentot* "*Venus.*"

"*Venus.*" We offer the sketch of this singular variety of our species, from a desire of promoting useful investigation, and shall feel ourselves obliged by the correction of any correspondent who can obtain better information on the subject. The person who exhibits this unfortunate female, either cannot, or will not give any further account of the place of her nativity, than that she was brought one thousand miles from the interior of Africa to the Cape.

As naturalists, we may be gratified with the sight of a being which differs in figure from any thing we have yet observed in human shape; but when we reflect that she is taken from her friends and her country, not for the purpose of receiving the blessing of religion, or the benefit of education; not for the purpose of enlarging our acquaintance with the human race, but is confined as a prisoner, and exhibited daily at so much a head, like an Orang Outang, or any other wild animal, to the gaze of an unfeeling multitude, we are ready to ask, is humanity become an empty name, and is the boasted liberty which the meanest slave is said to possess, the moment he treads the soil of this favoured country, for ever fled from amongst us?——The poet tells us,

> "Slaves cannot breathe in England; if their lungs
> "Receive our air, that moment they are free;
> "They touch our country, and their shackles fall."

The peculiar appearance of this woman at once fixes our attention, and suggests a query interesting to the naturalist, whether the protuberance of the nates is the consequence of some diseased action, or whether a distinct tribe of human beings exists, of which such a peculiarity of form characterizes the species? To ascertain this point, application was made by one of the Editors of this Journal, for permission to view her in her natural state; but this was refused, although it had been granted to persons whose rank in life and usual pursuits secured from the suspicion of being actuated by the love of science.

She is covered from head to foot with a sort of stuff which fits close to the body; but in her own country is said to be unused to any other covering than a few beads, a bracelet, and a small *apron of modesty.* Her height is four feet five inches: she is about 20 years of age, and has borne a child. The colour of the skin is tawny; the eyes are rather small, the eye-brows are painted black, and the forehead of a vermilion tint, whilst the cheeks are thickly dawbed with black and red. On her bosom hang two shells of the small land tortoise, containing paint; these she will not suffer to be handled. The lips are thick, resembling those of a negro; and the breasts breasts hang down low. The proportion of the arm is beautiful, the hands and fingers are small, delicate, and well turned. The thighs, legs, and feet also, are well formed, though short. She cannot speak any language intelligible to Europeans, neither does the man who exhibits her, and who was born at the Cape, seem able to converse with her except by signs. She walks or sits with ease, plays with her fingers upon a simple stringed instrument, and follows our fashion in diet. She seems melancholy, is possessed of sensibility, and at times appears indignant at the barbarous and degrading manner in which she is shewn before a gazing crowd, many of whom handle her pretty much as we see butchers when ascertaining the fatness of sheep and cattle in Smithfield market.

That the enlargement of the buttocks is not the effect of disease, seems probable, from the circumstance of the woman enjoying a good state of health, from her never expressing any sense of pain, or of uneasiness, on pressure being applied to the part; and from the facility with which she wields what appears to us an enormous and most aukward incumbrance.

Although several travellers have visited different parts of Africa, a very small portion of that extensive region has yet been explored. Our credulity has been abused with marvellous accounts of what they have seen, and these, succeeding voyagers have in so many instances ascertained to be false, that we are disposed to doubt any relation which is extraordinary, or which materially differs from the conceptions which we had previously formed. Thus, when among other wonderful stories which the interesting and amusing Vaillant has narrated in his travels in Africa, he described a tribe of people resembling the subject of this paper, his relation was deemed fabulous, and he even incurred from Barrow the reproach of having described the inhabitants, and the natural productions, as well as the geography of countries which he had never visited. His description of the Houzouanas, however, bears a striking resemblance to the female at present exhibiting in Piccadilly. He informs us, that they are a numerous people, lead a wandering life, passing great part of the year in making long journies; but that they possess a large tract of country extending from East to West, from Caffraria or Kafferland, to the country of the Great Nomaquois, or Namaaquas.

The Houzouanas are small in stature, the height of the men being about four feet and a half, five feet is reckoned tall. They are well proportioned, brave, active, indefatigable. The form of the cranium resembles that of the Hottentot, but the the chin is more round. They are not so dark in complexion, their hair is very short and curled; their nose flattened, little more than the nostrils being visible. The eyes are large and lively. They go almost entirely naked; are very insensible to changes of weather, and dwell in huts. They are humane and sociable; but from their erratic condition, exposed to frequent warfare with surrounding tribes. Their arms are bows and arrows. The women are distinguished by an immense projection of the posteriors, which Vaillant ascertained to consist of fat and fleshy fibres, which at every movement of the body, have a singular jumping undulating motion. When travelling they sometimes place a child upon the projecting buttocks. The hands and feet of the female Houzouanas are small and pretty, and their arms beautiful. They wear sandals, cover their heads with a jackal's skin, wear a small apron in front, and leaving the other parts of the body naked. They carry about them a little box, of wood, ivory, or tortoise-shell, which contains the grease with which they anoint themselves, and the tail of some animal, with which they wipe off the perspirable matter. Their manners are simple, and pleasing, but when insulted by enemies, or provoked by famine, they are ferocious and vindictive, and have often been mistaken for Bosjesmans. The only point of dssimilarity in this description is the size of the eyes. There is a tendency in most of the Hottentot women, as they advance in years, to form fat in the region of the nates, but the protuberance has never reached the extent which is observed in the subject of this paper, neither are the Hottentot women subject to it when young. Mr. Barrow altogether discredits Vaillant's account of the Houzouanas, but he describes the women of the Bosjesmans so distinctly, that there can be little doubt that he and the French traveller differed only in the name of the tribe, a circumstance very likely to happen, where neither of those gentlemen understood the language of the people where they visited. Barrow thus describes them.

"The great curvature of the spine inwards, and extended posteriors, are characteristic of the whole Hottentot race; but in some of the small Bosjesmans they are carried to a most extravagant degree. If the letter S be considered as one expression of the line of beauty to which degrees of approximation are admissible, these women are entitled to the first rank in point of form. A section of the body, from the breast to the knee, forms really the shape of the above letter. The projection of the posterior part of the body, in one subject, measured five inches and a half from a line touching the spine. This protuberance consisted of fat, and, when the woman walked, had the most ridiculous appearance imaginable, every every step being accompanied with a quivering and tremulous motion, as if two masses of jelly were attached behind.

In the present state of our knowledge of Africa, we cannot precisely determine the characteristic marks of every tribe which subsists in that vast country. In many instances, unquestionably, Barrow has disproved the assertions of Vaillant; but we do not think that the former writer has sufficiently explored the interior of Africa, or become so familiar with the customs and language of the inhabitants whom he met in the course of his excursions, to pronounce with certainty that the Bosjesmans whom he described in the preceding extract, were not the Houzouanas of Vaillant. At all events, it appears from their united evidence, that a people does exist with this singular form before alluded to; but one traveller refers them to one tribe, the other traveller to another tribe. From all then that has been stated by these authors, and from the appearance of the woman, at present in London, we think it may safely be concluded, that the protuberance is not the effect of disease, but is the characteristic of a distinct tribe of people.

