# Errata

**Published:** 1810-11

**Authors:** 


					388, line 14 from bott. for where read \vhom.

				

